# Rural Kenyan men's awareness of danger signs of obstetric complications

**Published:** 2011-11-14

**Authors:** Alice Dunn, Sayeed Haque, Michael Innes

**Affiliations:** 1 University of Birmingham, England

**Keywords:** Kenya, obstetric complications, men, knowledge, awareness, Africa

## Abstract

**Background:**

For many women in Kenya, their husbands act as gate-keepers to access of healthcare services. Awareness of the danger signs of obstetric complications is the essential first step in accepting appropriate and timely referral to obstetric care. The objectives of this study were to assess men's awareness of the danger signs of obstetric complications, and to identify any associated demographic factors.

**Methods:**

A cross-sectional study using a non-validated questionnaire was completed by 167 men with a wife or partner that had been pregnant in the last 36 months. The study took place in Muhoroni, in the Nyanza province of Kenya. Statistical comparisons were done using Kruskal-Wallis, Mann-Whitney and the Spearman's correlation coefficient.

**Results:**

Men displayed good knowledge of the danger signs of obstetric complications (median, 9/10; range 0-10; IQR, 7-10), with 92.2% (n=154) of participants recognizing severe abdominal pain, 91.6% (n=153) recognizing absence of foetal movement, and 90.4% (n=151) recognizing long labour as obstetric danger signs. More educated participants were significantly more knowledgeable than less educated participants (Kruskal-Wallis H=14.47; df=3; p=0.002).

**Conclusion:**

Participants were very proficient in identifying the danger signs of obstetric complications, with better knowledge in more educated men. Maternal mortality across Kenya remains high, however, so assessing knowledge nationally, and researching whether men are translating this knowledge into action would be recommended.

## Background

Childbirth is the time of greatest lifetime risk of mortality for a mother and her baby [1]. Each year, over 500 000 women and girls worldwide die of complications related to pregnancy and childbirth [2]. Ninety-nine percent of these occur in developing countries, and 50% in sub-Saharan Africa [3], an area being addressed by Millennium Development Goal (MDG) 5, which aims to have decreased the maternal mortality ratio (MMR) by 75% by 2015 [4].

Compared to the average MMR of 8 per 100 000 live births in industrialized countries, Kenya has an estimated MMR of 560 per 100 000 live births [5]. The main causes of maternal mortality, including haemorrhage, postpartum infection and hypertensive disorders [6,7], are all treatable. With awareness of the signs and symptoms of obstetric complications, and timely access to appropriate emergency obstetric care, many maternal deaths could be prevented, even in the poorest communities [8].

Evidence suggests that the majority of pregnant women in Kenya have access to antenatal care (ANC), with levels of access varying from 76-92% between different surveys [9]. Since the government's introduction in 2001 of a new model of ANC, recommended by the World Health Organization (WHO) [10], individual counselling about birth preparedness and danger signs of obstetric complications have been emphasized. Furthermore, those women identified as “at risk” for a complicated birth can be advised to give birth at a health-care facility or government hospital. Having a health worker with midwifery skills present at delivery is now seen as one of the most critical interventions for making motherhood safer [11].

However, the majority of Kenyan women have poor status in society and lack decision-making power, a factor that can contribute significantly to adverse pregnancy outcomes [2]. Various studies in Africa have found that husbands and other family members often make the decision about where a woman will deliver [11,12], and although it is unlikely that the men are actively ignoring the signs of complications during pregnancy and labour, it is possible that they lack awareness of what to look for, thereby hindering their ability to judge when emergency actions must be taken [13].

If men are acting as gate-keepers to women's health, it is paramount they are able to recognize the danger signs of obstetric complications [14]. The aim of this study, therefore, was to assess men's awareness of the danger signs of obstetric complications in a rural district in Kenya, and to identify any associations between knowledge and men's demographics. This information is of importance for targeted actions through antenatal care services in order to decrease maternal mortality.

## Methods

A cross-sectional study was carried out in Muhoroni, a rural town spanning an area of 2327 square kilometres in the Nyanza province of western Kenya. Inclusion criteria limited participants to willing, competent, adult males with a wife or partner who had been through childbirth in the preceding 36 months. Resource limitations led to those unable to speak English, Kiswahili or the local tribal language of Luo being excluded from the study. Participants were mainly recruited from the local sugar-cane factory, where men in various roles, each requiring different levels of education, were selected, and also from the mission hospital and around the town. All participants were approached directly by the researcher.

Data were collected between February and March 2010 using a questionnaire, designed by the researcher. It comprised two sections; the first, questions about participants’ demographics and exposure to pregnancy (how many pregnancies the participant's wife (wives) had experienced, and the date of the most recent), the second, a list 20 medical signs and symptoms, half associated with possible complications in a pregnant woman, half not (Additional material 1). The items for scoring were derived from two papers which had evaluated women′s knowledge of the danger signs of obstetric complications [14,15].

The questionnaire was piloted on 20 people before being amended and used. Because there was no gold standard and little work on men′s knowledge before, no validation of the scoring system was undertaken. Two methods of scoring were explored in the preliminary analysis to measure participants’ knowledge of the danger signs of obstetric complications. The first scoring system (henceforth ‘overall knowledge score’) awarded one point if a participant indicated ‘yes’ to a “true” obstetric danger sign, or ‘no’ to a “false” sign. Conversely, a point was deducted if a “true” sign was not identified or a “false” sign was believed to be related to obstetric complications. This gave participants a score between -20 and 20, where a score of 20 indicated that the participant had correctly identified all the “true” danger signs as well as all the “false” signs, and a score of -20 showed that the participant had mistaken every sign. Arguably, however, identification of a “false” sign does not pose any threat to a pregnant woman, yet missing a “true” danger sign does. Therefore a second score (henceforth “identification of danger signs alone”) simply awarded a point for each danger sign that was correctly identified. The relevance of these two scoring systems becomes apparent in the results section.

The questionnaire, participant information and consent documents were developed in English and delivered either by the researcher, in English, or a translator in Kiswahili or Luo. Each interpreter was trained about the aims of the project, the importance of confidentiality and accurate translation, and was required to sign a translator agreement.

The Internal Ethics Review Committee at The University of Birmingham, England, as well as the two town chiefs, one of whom was also a government representative, provided ethical approval for the study. Written consent was obtained from all participants after they had understood the importance of the research, the reason they had been approached, and that participation was voluntary and would be anonymous.

The primary aim of this study, assessing men's knowledge of the danger signs of obstetric complications, was calculated using proportions, and presented graphically. The secondary aim, whether there was any association between men's knowledge and their demographics, used Kruskal-Wallis test, Mann-Whitney U test and Spearman's correlation coefficient to make statistical comparisons. All data were analyzed using the statistical program SPSS, version 17.0 (SPSS Inc, Chicago, IL, USA).

## Results

One hundred and sixty-seven participants were recruited through purposive sampling for educational achievement. Their demographics are shown in Table 1. Of the participants, 20 (12%) had not completed primary school (education level 1), 38 (22.8%), had completed primary school (education level 2), 61 (36.5%), had completed secondary school (education level 3), and 48 (28.7%) had progressed to further education (education level 4). The high numbers (74.9%) of those reporting their tribe to be Luo is characteristic of the Nyanza province, where the majority of Luos reside.

**Table 1:** Demographic characteristics of the respondents

Characteristics (n=167)	Mean + standard Deviation or number (percentage)
**Age (years)**	35 + 8
	
**Education**	
Not completed primary school	20 (12)
Completed primary school	38 (22.8)
Completed secondary school	61 (36.5)
Further education	48 (28.7)
	
**Number of children**	
0-2	69 (41.3)
3-4	50 (29.9)
5+	48 (28.7)
	
**Tribe**	
Luo	125 (74.9)
Not a Luo	42 (25.1)
	
**Marital status**	
Married	160 (95.8)
Other	7 (4.2)
	
**Number of wives**	
0	4 (2.4)
1	151 (90.4)
2	12 (7.2)
	
**Religion**	
Christian	164 (98.2)
Other	3 (1.8)

**Figure 1:**
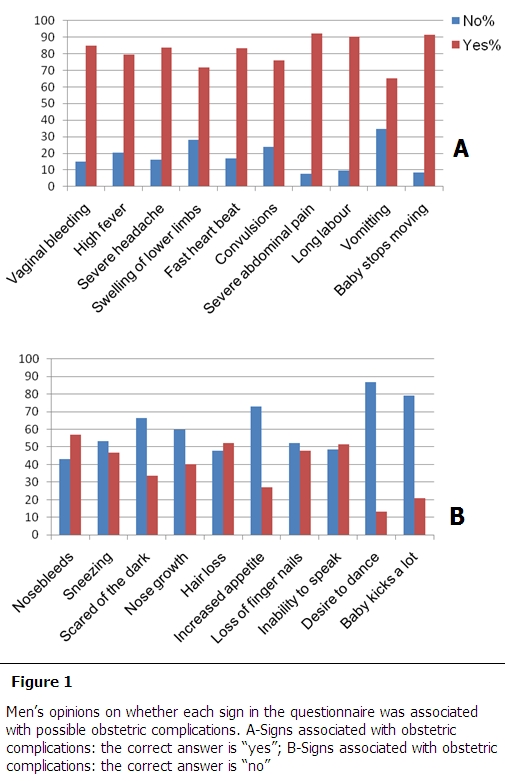
Men's opinions on whether each sign in the questionnaire was associated with possible obstetric complications. A-Signs associated with obstetric complications: the correct answer is “yes”; B-Signs associated with obstetric complications: the correct answer is “no”

Figure 1 illustrates the percentages of ‘yes’ and ‘no’ answers to each sign in the questionnaire. Severe abdominal pain (n=154, 92.2%), baby stops moving (n=153, 91.6%) and long labor (n=151, 90.4%) were the three most commonly recognized danger signs of obstetric complications. The desire to dance (n=145, 86.8%) and fetal movement (n=132, 79%) were the two “false” signs most recognized as such. Nose bleeding (n=95, 56.9%), hair loss (n=87, 52.1%) and inability to speak (n=86, 51.5%) were the “false” signs most incorrectly mistaken for a “true” danger sign.

Participants’ scores for their overall knowledge and the identification of danger signs alone are presented in Table 2 and Table 3, respectively. For the overall knowledge score, the median was 8 (range, -8 to 20; IQR, 6 to 12). Results were not normally distributed (Kolmogorov-Smirnov=0.104, p<0.0001). For identification of danger signs alone, the median score was 9, with over 75% of participants recognizing 7 or more danger signs (range, 0 to 10; IQR, 7 to 10). These results were also not normally distributed (p<0.0001).

**Table 2:** Overall knowledge score achieved by participants

Score^a^	Frequency	Percent	Cumulative Percent
−8	1	0.6	0.6
−2	2	1.2	1.8
0	8	4.8	6.6
2	12	7.2	13.8
4	12	7.2	21.0
6	23	13.8	34.7
8	30	18.0	52.7
10	24	14.4	67.1
12	27	16.2	83.2
14	16	9.6	92.8
16	5	3.0	95.8
18	6	3.6	99.4
20	1	0.6	100.0
**Total**	**167**	**100.0**	

^a^A score of 20 indicated that the participant had correctly identified all the “true” danger signs as well as all the “false” signs; a score of −20 showed that the participant had incorrectly mistaken every sign.

**Table 3:** Number of danger signs recognised by participants

Score	Frequency	Percent	Proportion (%) correctly identifying row score or above correctly^a^
0	1	0.6	100
1	1	0.6	99.4
2	0	0.6	98.8
3	3	1.8	98.8
4	6	3.6	97.0
5	12	7.2	93.4
6	9	5.4	86.2
7	13	7.8	80.8
8	24	14.4	73.1
9	43	25.7	58.7
10	55	32.9	32.9
**Total**	**167**	**100.0**	

^a^E.g. 58.7% correctly identified 9 or more danger signs

The two scores were then analyzed to identify any correlations with their educational level, number of children, tribe or age. The sample was too homogenous to analyze for marital status, religion or number of wives.

### Education

The median overall knowledge scores for those in education level 1 was 8 (range, 0 to 12; IQR, 6 to 10), for those in education level 2, 7 (range, -8 to 16; IQR, 4 to 10.5), for those in education level 3, 8 (range, -2 to 20; IQR, 6 to 12), and for those in education level 4, 10 (range, -2 to 18; IQR, 8 to 14).

There was a significant difference between the overall knowledge scores of participants in different educational levels (Kruskal-Wallis H=14.473; df=3; p=0.002), and significant differences between the scores of participants in education level 4 from all other education levels (level 1; Mann-Whitney U=424.500, p=0.001, level 2; Mann-Whitney U=537.500, p=0.001, level 3; Mann-Whitney U=1114.000, p=0.031). There was no significant difference between scores in different education groups for identification of danger signs alone (Kruskal-Wallis H=4.546; df=3; p=0.208).

### Number of children

A median overall knowledge score of 8 (range, -2 to 20; IQR, 6 to 12) in participants with 0-2 children was not significantly different from those with 3-4 children, whose median was 9 (range, -2 to 18; IQR, 6 to 12), or those with 5 or more children, whose median was 8 (range, -8 to 18; IQR, 4 to 12) (Kruskal-Wallis H=1.063; df=2; p=0.588).

Similarly, there was no significant difference between scores for identification of danger signs alone between participants with different numbers of children (Kruskal-Wallis H=4.841; df=2; p=0.89).

### Tribe

The median overall knowledge for those stating Luo as their tribe was 8 (range, -8 to 20; IQR, 6 to 12), which was not significantly different to those of the other tribal origins comprising of nine different tribes, whose median was also 8 (range, 0 to 18; IQR, 6 to 12) (Mann-Whitney U=2351.000, p=0.424). There was also no significant difference between scores for identification of danger signs alone between the tribes (Mann-Whitney U=2136.500, p=0.100).

### Age

There was no correlation between age and overall knowledge score (Spearman's correlation coefficient, 0.041; p=0.599) or identification of danger signs alone (Spearman's correlation coefficient, 0.017; p=0.828).

## Discussion

This is the first study done assessing men's awareness of the danger signs of obstetric complications in Kenya, and in this rural town, men, overall, had good knowledge on this subject. Over 90% of men were able to identify severe abdominal pain, lack of fetal movement and a long labor as a danger signs of obstetric complications, and the median score for the identification of the 10 obstetric danger signs was 9. They were, however, less able to identify signs not associated with obstetric complications, with over 50% of participants thinking that nosebleeds, hair loss and inability to speak were indicative of obstetric danger.

The high level of knowledge men appear to display in this study is not concordant with prior reports. Although research has not previously assessed men's knowledge of obstetric danger signs, studies in Kenya and other countries that have researched women's knowledge demonstrated a low awareness [14–16], potentially suggesting that men would have an equally, or even lower understanding. A study in Kenya showed that only 6.9% of women were able to identify three or more obstetric danger signs [14], yet 75% of men in this study were highly able to identify at least seven danger signs. The difference in knowledge observed is difficult to explain but may be due to the fact that in this study participants were prompted to recognize the signs through the questionnaire, rather than being asked to recall any signs from memory. If this is true, however, it is not perceived to be a weakness of this study, as men would also be prompted if their wife or partner was to experience any of the danger signs.

Men with a higher level of education were more aware of obstetric danger signs. This is in keeping with previous studies which have demonstrated that more educated women were more likely to be aware of danger signs and have better health outcomes than less educated women [14,15,17]. Similarly, a study in Nairobi, Kenya's capital city, showed that women with more highly educated husbands were more likely to deliver at a healthcare facility with trained personnel, rather than at home, which is now seen as one of the most important interventions for making motherhood safer [18]. It is perhaps not surprising that education is important for understanding health messages and to be able to make decisions regarding healthcare [15].

However, despite their good knowledge about obstetric danger signs, participants were less able to identify signs not associated with obstetric complications. One limitation of this study is the language barrier, and it is possible that less educated participants had difficulty in comprehension of the concept “medical problem associated with pregnancy”, as opposed to a medical problem in general when answering the questionnaire. This could be a potential explanation for the fairly high numbers of incorrect answers to the “false” danger signs, especially from the less educated participants, who were significantly less able to identify these signs compared with the more educated participants (Kruskal-Wallis H=14.473; df=3; p=0.002). It could also explain the higher number of incorrect answers to “nosebleeds”, “hair loss” and “inability to speak”, which could infer medical complications, rather than “desire to dance” and “baby kicks a lot”, two things that would probably be considered medically normal. An attempt to control this was made by training the interpreters during the piloting of the study and allowing them to practice the best way of conveying the meaning of the questionnaire without coercing participants into the correct answer. It must also be re-emphasized that despite the fairly high numbers of men mistaking “false” signs for danger signs, participants were still adept at identifying the correct danger signs (median, 9; IQR, 7-10), which is reassuring.

Responses may have been affected by social acceptability bias. If in doubt of the answer, participants may have been inclined to tell the researcher that a sign was indicative of obstetric danger, to prevent feeling embarrassed about getting an answer wrong and missing an important sign. However, despite assuring participants at the beginning of the interview that they did not have to answer all questions, 98% of questionnaires were completed fully. Furthermore, it was pointed out in the questionnaire that there were 10 signs that were related to obstetric complications, and 10 that were not. Lastly, only two men of the 169 approached refused to take part, suggesting participants were not afraid of not being able to answer the questions.

It was expected that there would be increased awareness among older participants, and participants with multiparous wives, reflecting personal experience or community events. It was surprising; therefore, that knowledge did not increase with either. It does suggest, however, that education, above all other factors, is key in influencing men's knowledge of the danger signs of obstetric complications.

## Conclusion

Men in the rural town of Muhoroni were predominantly able to identify the danger signs of obstetric complications, although were less able to recognize signs not associated with obstetric complications. The ability to distinguish between “real” signs and “false” signs increased significantly with higher levels of education. However, despite men's apparent high knowledge of the danger signs of obstetric complications, there is still a high maternal mortality ratio across Kenya, and in much of Sub-Saharan Africa. Similar research needs to be done to assess men's knowledge nationally, but, importantly; research also needs to be done as to whether men are able to translate their knowledge into beneficial behaviour, allowing their wives and partners to access modern obstetric care.

## Additional material

### Additional material 1

Questionnaire used by participants: http://www.panafrican-med-journal.com/content/article/10/39/annex/39_1.pdf (PDF 55Ko)
